# Abstract of the Proceedings of the Buffalo Medical Association

**Published:** 1863-06

**Authors:** J. F. Miner

**Affiliations:** Secretary


ART. III.—*Abstract of the Proceedings of the Buffalo Medical Association.*

TUESDAY EVENING, May 5th, 1863.

*Dr. White* presented a specimen of extra uterine pregnancy which had been sent him by Dr. Lauderdale. The uterus and appendages, together with the child placenta and cord had all been carefully removed and forwarded entire. The history of the case had not yet been fully furnished, and therefore could not at this time be presented in detail. The child weighed thirteen pounds, the whole uterus, placenta and fœtus seventeen pounds, and the mother regarded her term of gestation as having been prolonged nearly or quite two months, making it eleven months from the time of her last menstrual secretion until the time of the rupture of the membranes and sac, and death of both mother and child. The uterus was enlarged to the usual size of that organ at the third month of pregnancy. One fallopian tube was attached nearer the fundus than the other, and the one lowest upon the side of the organ had the immense dilatation which had been caused by the gradual development of the fœtus, the fimbriated extremity remaining perfectly distinct; the point of development being from within the fallopian tube, constituting what is usually denominated tubal pregnancy. The specimen had been preserved some time in alcohol, and the minute anatomical structure of the membranes or coverings of the fœtal cyst could not be accurately determined, though that it was tubal, rather than tubo-ovarian or tubo-abdominal, was quite evident. Tubal pregnancy continuing up to the full period of gestation, was so far as he was informed exceedingly rare; on this account the specimen presented was of great interest. This was perhaps the most frequent of all the varieties of extra-uterine pregnancy, and very rarely continued beyond the second or third month, before rupture of the tube was produced, and fatal peritonitis.

*Dr. Ring* remarked upon the treatment proper in such cases as had been presented by Dr. White. Thought also that in cases of rupture of the uterus, cæsarean section should be made and the child immediately removed, certainly if delivery could not be otherwise effected, and was not sure but it would be better to perform this operation in many cases even if the delivery could be effected, for the purpose of removing the blood, &c. from the abdominal cavity. Hæmorrhage might perhaps be arrested by ligating the vessels. Some way should be devised of saving women after this accident.

*Dr. Samo* inquired about the symptoms of extra-uterine pregnancy; if there was usually plain evidences of anything abnormal, especially during the early months; also if the location of the tumor could lead to any opinion of the development being extra-uterine.

*Dr. White* replied at length to these inquiries, and thought it could no throw much light upon it, the tumor being so different in different cases. Dr. W. thought that by use of sound and all the means of diagnosis at our command, the condition of the female from which this child was removed could have been quite certainly determined, sufficiently so to have justified operation for removal of the child at the moment of rupture or pain indicating rupture. Still physicians would act wisely in using great care in making diagnosis before resorting to operative interference.

*Drs. Wyckoff and Rochester* spoke of cases of pregnancy, where the abdominal tumor fell over so much in front as to necessitate its being held up out of the way by assistants; this showing that the location of abdominal tumor in pregnancy could throw little if any light upon its being extra-uterine or otherwise.

*Dr. Rochester* wished to mention a matter which should go on record; hoped Dr. Gould would have been present to have presented the subject; had been invited to see the case by Dr. G. A man styling himself Dr. Walter Crandall of Alden, had attempted abortion upon the woman by introducing sponge far up into the os uteri. The woman was three or four months pregnant, and had heard that this man gave medicine to procure abortion. She wrote to him for the medicine, but instead of this he visited her. Told her in hearing of a lady friend that he had often relieved women by use of an instrument, and finally persuaded her to allow the operation. She said his operation hurt her greatly. The woman grew severely sick, and Dr. Gould was called; the sponge was removed the third or fourth day. She was properly treated and was recovering. The uterus had not yet thrown off its contents.

This case was referred to with the view that something should be done to bring this crime to punishment. The probabilities were that since the woman had recovered no farther action would be taken in the matter, and the villain practicing such rascality would be left to procure another victim. Had introduced the subject knowing that something should be, and thinking that possibly something might be done to prevent such crime.

*Dr. White* said he had formerly, in connection with Drs. Flint and others, attempted to influence legislation upon this subject, hoping to lessen this crime. Thought that it was not so much the want of proper legislation, as lack of perception upon the part of the people of the aggravated character of this sin. All the influences had been directed and brought to bear upon men styling themselves physicians, while we want the public educated to regard producing abortion, as they now regard infanticide and murder. The men who now do it, would do it, if hanging was the legal penalty; they are below all moral influences, and ready to commit the most revolting crimes. Is not able to know where or how to commence the reform. The evil is a growing one, and has thus far resisted all efforts to control it.

*Dr. Wyckoff* remarked upon the frequency of application for having abortion produced, and the apparent feeling that it was proper and desirable, not once thinking it was a crime. He thought females generally regarded it as quite innocent in themselves, even though it might be a misdemeanor in their estimation in any one assisting them; still it rarely occurred to them, that it was a crime to procure abortion, and certainly not if done at an early period of gestation.

*Dr. Ring* wished to relate how he treated these applications. He takes time and pains to tell these patients the true character of this crime; and does not unceremoniously dismiss them, but tries to give them correc notions concerning it.

*Dr. Rochester* spoke of the prevalence of roseola, and thought the Secretary would recollect some time since, about the first appearance of the disease in Buffalo, how he expressed doubts about the communicability and diagnosis of the disease, and would like to know if his opinions had in any way changed since that time, or if his experience since had led to the belief of its communicability? Spoke also of measles, and of the symptoms and means of differential diagnosis between these two diseases. Had seen several cases in those who had previously had measles. Dr. R. further stated that he presumed the affection is the same as described under the title of Rubeola sine Catarrho, but that it was not properly a rubeolus disease, inasmuch as it did not protect against subsequent attacks of measles, nor did an attack of severe true rubeola, afford any immunity against the disorder under consideration.

*Dr. Miner* remarked that he had since seen considerable many cases of the disease referred to by Dr. Rochester, and that in many cases it certainly appeared to be communicable; that it was or was not, he had no opportunity of determining, but many cases seemed to have been contracted by exposure to the disease. At the time referred to by Dr. Rochester, he took the definition of roseola to be correct as generally given by authors upon skin diseases, "A rose-colored efforescence, variously figured, without wheals or pimples, and not contagious." All authors appear to regard the disease in its various forms as non-contagious and non-infectious, and he had no proof that they were incorrect. The disease which has been so prevalent in Buffalo has appeared in many instances to be communicable, and physicians and families have no doubt upon this point. Many physicians have called this disease measles, and seemingly observed nothing unusual in the form of it. If it is established to be contagious, it will then appear highly probable that the disease is not properly speaking roseola, but the mild form of rubeola, denominated by Wilson and others "*Rubeola sine catarrho*," which is spoken of as identical with rubeola vulgaris, with the exception of the catarrhal and febrile symptoms, which are either exceedingly mild or wholly absent. This disease also prevails during epidemics of measles and children affected with this form are more liable to a second attack of measles than those who have experienced an attack of the ordinary kind.

*Dr. White* proposed for membership Dr. Charles Winne.

*Voted*, on motion of Dr. Rochester, that Dr. Ross, House Physician and Surgeon to the Buffalo General Hospital, be invited to participate in the transactions of the Society.

*Voted* to adjourn to the first Tuesday evening in June.

J. F. MINER, Secretary.

